# Effects of combined training during the COVID-19 pandemic on metabolic health and quality of life in sedentary workers: A randomized controlled study

**DOI:** 10.3389/fpubh.2022.1040714

**Published:** 2022-11-10

**Authors:** Fernanda M. Silva, Pedro Duarte-Mendes, Eugénia Carvalho, Carlos M. Soares, Carlos Farinha, João Serrano, Rui Paulo, Alain Massart, Rafael N. Rodrigues, Ana M. Teixeira, José Pedro Ferreira

**Affiliations:** ^1^Faculty of Sport Sciences and Physical Education, University of Coimbra, Coimbra, Portugal; ^2^Research Unit for Sport and Physical Activity (CIDAF, UID/DTP/04213/2020), University of Coimbra, Coimbra, Portugal; ^3^Department of Sports and Wellbeing, Polytechnic Institute of Castelo Branco, Castelo Branco, Portugal; ^4^Sport, Health & Exercise Research Unit (SHERU), Polytechnic Institute of Castelo Branco, Castelo Branco, Portugal; ^5^Center for Neuroscience and Cell Biology, University of Coimbra, Coimbra, Portugal; ^6^Institute for Interdisciplinary Research, University of Coimbra, Coimbra, Portugal

**Keywords:** physical activity, insulin resistance, stress-reducing interventions, COVID-19, quality of life

## Abstract

This study aimed to analyze the effects of a combined training (CT) program performed during the first national lockdown due to the COVID-19 pandemic on body composition, metabolic profile, quality of life and stress in sedentary workers, and examines whether changes in the metabolic profile are associated with changes in health-related outcomes which are modifiable by exercise. We evaluated 31 sedentary workers (48.26 ± 7.89 years old). Participants were randomly assigned to a CT group (i.e., performed 16 weeks of exercise) or to a non-exercise control group. The CT program consisted of 16-week of resistance and aerobic exercise. Body composition, glycemic and lipidic profiles, cardiorespiratory fitness (CRF), health-related quality of life and stress levels were assessed pre- and post-intervention. After the intervention period, the CT group demonstrated significantly lower waist and hip circumference (*p* < 0.05) values than the control group. The control group significantly increased the fasting glucose and HOMA-IR after 16 weeks follow-up (+4.74 mg/dL, *p* = 0.029; and +0.41 units, *p* = 0.010, respectively), whiles no significant changes were observed in the CT group in the same parameters (+3.33 mg/dL, *p* = 0.176; and +0.04 units, *p* = 0.628, respectively). No changes were observed in the lipid profile for either group (*p* > 0.05). A significant positive relationship was detected between the change in BMI with the changes in insulin and HOMA-IR (*r* = 0.643, *p* = 0.024; and *r* = 0.605, *p* = 0.037, respectively). In addition, the changes in CRF were negatively associated with the changes in total cholesterol (*r* = −0.578, *p* = 0.049). We observed differences between groups on perceived stress levels and physical, psychological, and environmental domains of quality of life, with the CT group showing better results. Moreover, the CT group improved perceived life satisfaction (+3.17 points, *p* = 0.038). The findings of the present study suggest that the participants who remained physically active during the first pandemic-related lockdown were able to mitigate the deleterious effects associated with a sedentary lifestyle.

## Introduction

Before the outbreak of the Coronavirus Disease 2019 (COVID-19) pandemic – an infectious disease caused by the severe acute respiratory syndrome coronavirus 2 (SARS-CoV-2) virus - European adults spent on average 8.83 h/day in sedentary behaviors, and 72% did not meet the recommended 150 min/week dose of moderate-intensity physical activity (PA) (1). Evidence shows that the occupational category has a significant impact on daily PA levels, with desk-based workers presenting the lowest number of steps and the higher sedentary time both at work and during awake hours (2, 3). After the World Health Organization (WHO) declared (on March 11th, 2020) the novel coronavirus outbreak as a pandemic, governments of the mainly affected countries implemented lockdowns and/or requested nationwide stay-at-home orders to counteract the spread of the virus (4). Furthermore, many working adults were required to work remotely from home (i.e., whenever the functions in question allowed it to stay safe) (5). Studies report that during the COVID-19 pandemic, due to lockdowns and working remotely, physical inactivity and sedentary behaviors were greatly exacerbated, in the adult population (5–7). These results have had grave health implications, particularly since prolonged sedentary behavior and physical inactivity are major risk factors for obesity, insulin resistance, and type 2 diabetes mellitus (T2DM) development (8–12). A sedentary lifestyle is associated with unfavorable changes in body composition with loss of muscle mass and accumulation of body fat (BF), mainly abdominal fat. This in turn stimulates chronic low-grade systemic inflammation and an increase in the prevalence of related comorbidities including insulin resistance and T2DM (9, 13). Furthermore, these behaviors have also been associated with poor mental health and wellbeing, including reduced health-related quality of life (HRQoL) (14, 15) and higher levels of stress (16, 17), among adults. It is vital to emphasize that the COVID-19 pandemic itself has become a threat to psychological health, due to stressors such as physical inactivity, quarantine/lockdowns, economic and financial instability, and fear (18).

Effective stress-reducing interventions such as, regular exercise are key to diminishing the deleterious impact of sedentary behaviors and physical inactivity on health outcomes (19, 20). Regular exercise is a cornerstone in the prevention of chronic non-communicable diseases, including metabolic disorders, since it induces metabolic and immunological health benefits (13, 21). In non-pandemic contexts, studies show that combined training (CT) (i.e., aerobic and resistance exercise) is an important tool to ameliorate levels of abdominal obesity (22–24), insulin resistance (22, 25–28), total cholesterol, low-density lipoprotein (LDL), and triglycerides (26, 27), among adults. However, these studies included subjects with different biological characteristics (i.e., elderly) (22) or with associated comorbidities (i.e., metabolic syndrome or T2DM) (22, 25, 27). Moreover, although studies have shown the benefits of regular PA in stress regulation and quality of life improvements (16, 17), the effectiveness of a specific CT program on these outcomes is unknown, in middle-aged adults.

According to Narici et al. (20), although the exercise to dose-response relationship is currently unknown, it appears that low-to-medium intensity exercise, even implementable in home-settings, will promote important health benefits. To our knowledge, this is the first experimental study to assess whether middle aged adult workers, who remained active (i.e., through a CT program supported by digital solutions) during the first wave of COVID-19 in Portugal, were able to mitigate the deleterious effects of sedentarism on body composition, metabolic profile, subjective quality of life and stress. Therefore, the main purpose of this study was to evaluate the effects of a CT program performed during the first national lockdown, due to the COVID-19 pandemic, on body composition, metabolic profile (i.e., glycemic and lipid profiles), quality of life and stress, in sedentary workers. In addition, we aimed to assess whether changes in the metabolic profile are associated with changes in health-related outcomes, which are modifiable by exercise [i.e., cardiorespiratory fitness (CRF), body composition]. We hypothesized that the workers who kept active through the CT program, during an adverse context of the COVID-19 pandemic-related lockdown, were not metabolically affected and presented a better perception of HRQoL and lower levels of stress compared to workers who maintained their usual sedentary habits.

## Methods

### Experimental approach

This study was designed as a 16-week randomized controlled trial (RCT) with parallel groups and it follows the Consolidated Standards of Reporting Trials (CONSORT) guidelines (29). The intervention was carried out over a period of 16 weeks (between January and May 2020). Following the baseline assessments, the participants were allocated into two groups with a 1:1 allocation, using a computer-generated simple randomization software: (a) an experimental group that performed 16 weeks of a CT program and, (b) a control group that maintained their current lifestyle (i.e., sedentary lifestyle), including no engagement in any structured exercise program. This randomization process was generated by an independent biostatistician. The study participants were enrolled and assigned to their respective groups by the principal investigator (participants were notified by e-mail or telephone). All participants were instructed to maintain the same dietary intake and daily PA levels over the intervention period. The result of randomization process was blinded to the research team responsible for carrying out body composition and CRF assessments to minimize the risk of bias.

**Figure 1:**
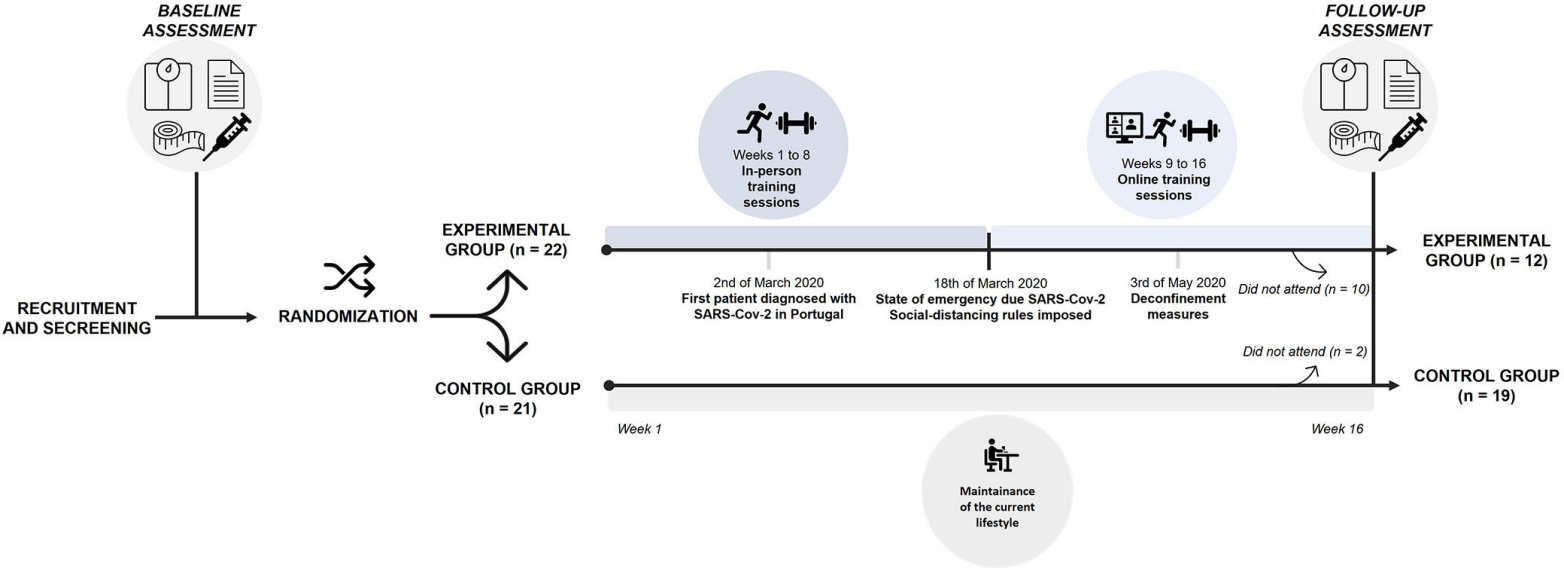
Study design with critical time points and tasks.

Assessments were performed at baseline (pre-intervention, before the implementation of social distancing rules) and 16 weeks later (post-intervention, performed at the end of the first pandemic lockdown), in both groups. It involved the following: biochemical assessment (fasting blood and salivary samples were collected), body composition, PA levels, dietary patterns, CRF, and HRQoL. At the end of the intervention program, 16 weeks later, the experimental group assessment was performed 72 h after the last exercise session, to prevent possible residual effects. Data and sample collection were carried out by invited specialists (nurses, health technicians) and co-investigators of the research team. The same testing staff performed the data and sample collection in the same order at baseline and 16 weeks later. The training program performed by the experimental group underwent some changes due to the unexpected first pandemic lockdown by COVID-19 in Portugal. Thus, the exercise sessions were performed in person until the eighth week (weeks 1 to 8), and in the following weeks (weeks 9–16), the training sessions were carried out online *via* the ZOOM platform (this period corresponds to the duration of the first lockdown due to the COVID-19 pandemic). Figure 1 presents the study design with the critical time points and tasks identified.

### Participants

A total of 54 participants with sedentary occupations were recruited and assessed for eligibility. Posters and flyers were disseminated to the busiest places in the workplaces, so that employees were aware of the study. The interested subjects contacted the research team, and an appointment was set up for a more extensive interview, during which the subjects were pre-screened for initial eligibility criteria. The possible eligible individuals were then invited to an in-person screening visit, where they signed an informed consent form and completed other screening measurements. Subjects that met the eligibility criteria returned for the baseline assessments. The inclusion criteria were as follows: [i] working adults with sedentary occupations (i.e., report spending ≥ 65% of their workday in sedentary behaviors); [ii] low levels of moderate-to-vigorous PA (30); [iii] having no chronic metabolic disease, cardiovascular disease, cancer, or other major illness; [iv] having no cognitive or psychiatric conditions that could interfere with the study outcomes; [v] no participation in any exercise program in the 6 months prior to screening; [vi] willingness to maintain the same dietary intake and participate in all of the study's procedures. We determined the sample size necessary for each group using the G^*^Power software (version 3.1.9.2, University of Kiel, Germany). For a medium effect size of 0.30, a sample size of 12 participants in each group (CT group vs. control group) achieves 80% power (β = 0.80) to detect significant differences within and between groups using an *F* test (α-level of 5%). More participants were recruited due to possible participant lost to follow-up. We also determined the sample size necessary for the association analysis, that results in a sample size of 12 participants (effect size *d* = 0.55; α = 0.05; statistical power = 0.65).

**Figure 2:**
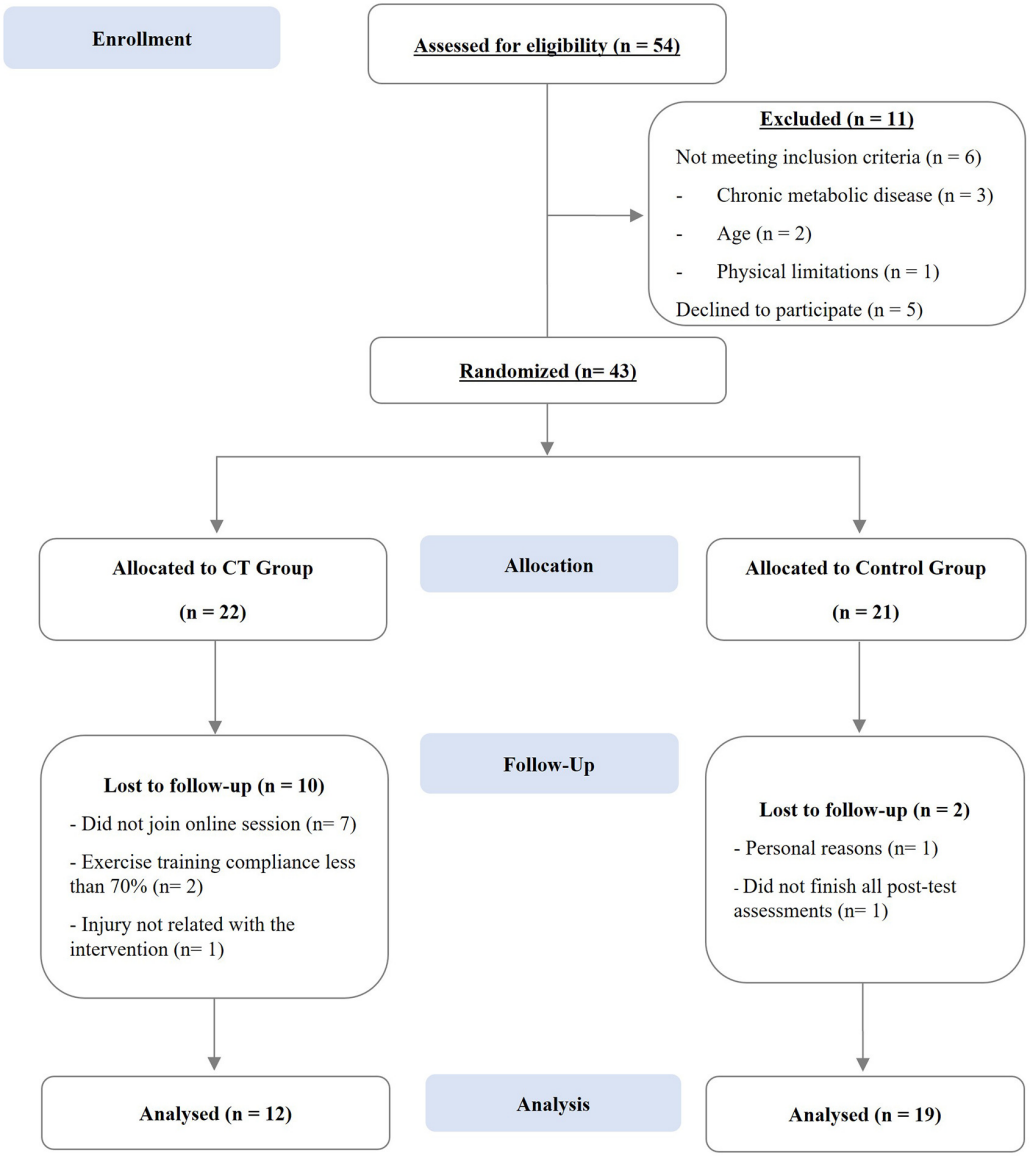
CONSORT diagram of study participants through each stage of the study.

Only 43 of the 54 participants who took part in the first screening met all the eligibility criteria and agreed to take part in this study. Participants who met all eligibility criteria were randomly assigned to either the CT group (*n* = 22) or the control group (*n* = 21). However, only 31 participants (48.13 ± 7.68 years old) of both genders completed the study (CT, *n* = 12; control group, *n* = 19). The reasons for withdrawal in the CT group were (a) did not participate in the online training sessions (*n* = 7); (b) failure to comply with over 70% of frequency during the whole training program (*n* = 2); and (c) injury unrelated to the intervention (*n* = 1). In the control group, 2 participants withdrew from the study for personal reasons or for not finishing all post-test assessments. A CONSORT diagram is shown in Figure 2. All participants included in this study signed a written informed consent, which complied with the recommendations of the Declaration of Helsinki (31) and was approved by the Ethical Committee for Health of the Faculty of Sport Sciences and Physical Education, University of Coimbra (reference: CE/FCDEF-UC/00512019).

### Exercise training

Attendance at sessions was recorded and entered on a database. To be integrated into the analysis, attendance of at least 70% of exercise sessions was required. The CT program was conducted following the American College of Sports Medicine (ACSM) guidelines (32, 33). The exercise sessions took place 3 times per week on non-consecutive days (i.e., Tuesday, Thursday, and Saturday), with duration of ~ 55 min/session, over 16 weeks. As mentioned above, in the first 8 weeks of intervention (phase I, weeks 1–8) the exercise sessions were held in-person in an enclosed gymnastics pavilion. In the following 8 weeks (phase II, weeks 9–16), the exercise sessions were performed online through the Zoom platform. This led to some adaptations in the training program. All exercise sessions were conducted and supervised by 2 instructors who graduated in Sport Sciences. Initially, the participants of exercise program were provided with two exercise sessions for familiarization with the exercise techniques and load of the free weights. After this period of familiarization, the maximal dynamic strength tests for determining training load were performed. Next, the training program carried out in the phase I (before the pandemic-related lockdown) and in the phase II (during the pandemic-related lockdown), will be described.

#### Phase I (weeks 1–8): In-person training sessions

The main part of each session included resistance training followed by aerobic training exercise. The resistance training lasted ~25 min per session and included seven workout stations/exercises. The resistance exercises (chest press, incline or flat push-ups, bent-over two-arm row, abdominal exercises (i.e., regular plank), front squat, calf raises, glute bridge) were performed with free weights or own subjects' body mass. Participants completed 10–15 tempo-controlled repetitions of each exercise in a 60-s period followed by 30-s of interval rest, before going on to the next exercise. Two rounds of the seven exercises circuit, with a recommended interval rest of 60–90 seconds, were completed in each session for resistance training. For the free weight exercises, the load was adjusted individually to work between 50–75% of the estimated 1-repetition maximum (1RM). A gradual progression in intensity was followed throughout the weeks (i.e., weeks 1–4, 12–15 tempo-controlled repetitions at 50–65% of 1RM, and rate of perceived exertion (RPE) of 5–6; weeks 5–8, 10–12 tempo-controlled repetitions at 60–75% of 1RM, and RPE of 5–6). Once an exercise could be performed comfortably in two consecutive training sessions, an ~5% increase in weight lifting was added to ensure that a progressive overload was provided. Participants were instructed to inhale and exhale during the eccentric and concentric phase, respectively. The aerobic training lasted ~15 min per session and involved fast walking and running with an intensity at 60–80% of the participant's maximum heart rate (HRmax). The aerobic training followed a gradual increase in intensity throughout the weeks (i.e., weeks 1–4, 60–70% HRmax, and RPE of 5–6; weeks 5–8, 70–80% HRmax, and RPE of 6–7). To assess and monitor the heart rate intensity interval, the participants used the Polar FT7 (Polar Electro Oy, Finland) monitor during all sessions. The target HR intensity was indirectly predicted by applying Karvonen's formula (34). HRmax was determined using the equation proposed by Gellish et al. (35). The resting HR was reassessed after 4 weeks so that target HR prescriptions could be continuously updated. To ensure that participants were exercising at the planned intensity, the intensity throughout the exercise sessions was also monitored through the RPE, using the Borg CR-10 scale (36). Each exercise session started with a dynamic standardized warm-up at a light-to-moderate intensity to increase body temperature (10 min) and finished with a cool-down period with aerobic activities of light intensity, and static stretching movements (5 min). All participants showed good exercise tolerance and none reported any injury or other major health problems related with the exercise intervention (i.e., injury or other).

#### Phase II (weeks 9–16): Online training sessions

With the lockdown and inherent policies imposed by the Portuguese government on March 18, 2020, the training sessions moved to an online format. This was the only way to proceed with the exercise program. As a result, the training program experienced various alterations, since participants did not have free weights adjusted to their capacities, nor did they have sufficient vast areas in their homes. For resistance training, participants did mostly the same exercises as in phase I, however, for exercises that required external loads, materials accessible to all participants were used, such as water bottles (5 L and 1.5L), milk packaging, etc. Due to these adaptations, it was not possible to continue to monitor the training intensity using the estimated 1RM. Thus, resistance training intensity was measured only through the RPE scale. The participants performed 12–15 tempo-controlled repetitions of one exercise in a 60-s period (RPE of 5–7 as a target intensity for each exercise), followed by 30 s of rest before advancing to the next exercise, respectively. A total of 2 rounds of the seven exercises circuit, with a recommended interval rest of 60–90 s, were performed in each resistance training session. The aerobic training also follows a circuit training methodology. In this way, 2 rounds of the 5–6 exercises circuit were completed in each session for aerobic training. The working time in each exercise was 40 s in the weeks 9 to 12 (RPE of 6–7) and 60 s in weeks 13 to 16 (RPE of 7–8). The resting times between workout exercises was 20 s in the first 4 weeks (weeks 9–12) and 30 s in the following weeks (weeks 13–16). Some of the aerobic exercises performed were jogging in place, jumping jacks, modified mountain climbers, etc. Appropriate adaptations were given to participants with more difficulties. RPE values were collected immediately after each exercise.

### Procedures

The baseline and the 16-week follow-up assessments were organized on 2 days, respectively. On day one, fasting blood and saliva samples were collected in the laboratory. On day two, body composition, CRF, HRQoL and the dietary pattern were assessed. The body composition and CRF were measured in a large room in both assessment moments. The follow-up assessment took place after the end of the lockdown and was carried out by a marking system, i.e., evaluation of 2 subjects per hour. All the participants and the research team wore surgical masks and disposable gowns to prevent the risk of contamination by the virus SARS-CoV-2. The research team also ensured the disinfection of all material used.

#### Anthropometry and body composition

Body mass (SECA 761, Germany) and height (Seca Bodymeter 208, Germany) were measured in duplicate using the standard protocols (37). The body mass index (BMI) was calculated dividing the body mass (in kg) by stature in square meters (kg/m^2^). Waist circumference (WC) and hip circumference (HC) were taken twice using a flexible steel tape (Hoechstmass-Rollfix, Germany) with an accuracy of 0.1 cm (38). These measures were used to calculate waist-to-hip ratio (WHR) and waist-to-heigh ratio (WHtR). Skeletal muscle mass (SMM), fat mass (FM), total BF, and fat free mass (FFM) were measured by a tetrapolar bioimpedance (Inbody 270, USA), following the standardized protocols and manufacturer' procedures (39).

#### Venous blood sampling

Blood samples (10 mL; venous) were collected in a seated position from the antecubital vein after a 12-h overnight fast into dry tubes and into tubes containing ethylenediaminetetra-acetic acid. Participants were reminded to maintain a hydrated state and to avoid strenuous physical efforts for 24 h prior to the collection. The tubes were refrigerated for 1 h before centrifugation for 10 min at 1,500 rpm at 4°C. Next, the serum and plasma content were stored at −80°Celsus (C), until the study was concluded, so that all samples could be analyzed together. Serum samples were used to analyze the lipid profile and glucose metabolism of the study participants. This included quantification of total cholesterol, high-density lipoprotein (HDL-C), triglycerides, and fasting glucose using the standard enzymatic assays (ABX Pentra, Germany). Fasting insulin concentration was assessed employing enzyme-linked immunosorbent assay (ELISA) (Crystal Chem, USA) according to the manufacturer's protocol. The LDL-C was determined using the Friedewald et al. (40) formula:


$$\begin{array}{lll} \text{LDL}-\text{C}\left(\text{mg}/\text{dL}\right) & = & Total\;cholesterol-\text{HDL}-\text{C}-\left(\text{Triglycerides}/5\right) \end{array}$$

Fasting insulin and glucose concentrations were used to calculate the homeostatic model assessment (HOMA-IR) using the following equation (41):


$$\begin{array}{l} HOMA-IR=\frac{fGlucose\;\left(mg/dL\right)\;x\;fInsulin\;\left(uU/L\right)}{405} \end{array}$$

A person is classified as insulin resistant when their HOMA-IR was >2.0 (42, 43). The blood sample collections (at baseline and at the 16 weeks follow-up) occurred at the same time in the morning (between 07:30 a.m. and 09:30 a.m.) in the laboratory.

#### Saliva samples

Saliva samples were taken from the study participants in a seated position by the passive drool method (44). Participants were reminded to maintain a hydrated state and to avoid alcohol drinks for 12 h, physical efforts for 24 h, brush teeth for 1 h, and consume foods with high acidity and/or sugar immediately before sampling (45). Moreover, 10 min before taking the saliva, participants were instructed to wash their mouth for 1 min with water to remove any food residues (45, 46). The polypropylene tubes were weighed before use. Once collected, saliva samples were immediately weighed and stored frozen at −20°C for posterior analysis. On the day of the analysis, samples were centrifuged for 4 min (13.0 × g) to remove particulate matter. Salivary levels of cortisol were analyzed by ELISA (Salimetrics, USA). The sensitivity and range of detection limits for cortisol were < 0.007 and 0.012–3.000 μg/dl. The α-amylase was analyzed by a kinetic reaction assay (Salimetrics, USA), according to the manufacturer's instructions. The saliva sample collections (at baseline- and 16 weeks later) occurred at the same time in the morning (between 08:00 a.m. and 10:00 a.m.) in the laboratory to minimize circadian effects.

#### Cardiorespiratory fitness

Maximal oxygen consumption (VO_2max_) was predicted using a valid and reliable submaximal step test – Chester Step Test (CST) (47, 48). The VO_2max_ predicted by the CST showed a strong and positive association with the VO_2max_ determined by a cardiopulmonary exercise test (*r* = 0.989) (49). The CST is a multistage test and starts with a very slow step rate of 15 steps/min, and every 2 minutes the HR and RPE are checked and recorded, in addition, the stepping rate is then increased slightly (47). The test stopped when the participants reached 80% of their HRmax (estimated by 220-age) or/and reports moderately vigorous level of exertion (RPE = 14) (47). One of our participants did not meet the CST requirements (i.e., complete at least 2 levels) and was therefore excluded from the VO_2max_ analyses. VO_2max_ (mlO_2_/kg/min) was determined using the Graphical Datasheets (47).

#### Subjective HRQoL

The brief version of the World Health Organization Quality of Life (WHOQOL-BREF) questionnaire (50, 51) was used to assess the study participants subjective quality of life. This questionnaire included 24 items (responses on 5-point Likert scale), in addition to 2 more that assessed the overall quality of life and health. This tool included 4 quality of life domains: physical health (7 items), environmental factors (8 items), social relationships (3 items), and psychological health (6 items) (51). Items 3, 4 and 26 were inverted to calculate the final score. Higher scores correspond to a better perception of quality of life (51). At baseline, there was sufficient internal consistency in the separate domains: physical (α=0.81), psychological (α = 0.79), social (α = 0.68) and environmental (α = 0.68). At the follow-up the results also show sufficient internal consistency in the different domains: physical (α = 0.82), psychological (α = 0.83), social (α = 0.42) and environmental (α = 0.80).

The Satisfaction with Life Scale [SWLS; (52, 53)] was used to assesses the participant's global judgment of life satisfaction. This scale comprised a 5-item (responses on a 5-point Likert scale; 1 = strongly disagree to 5 = strongly agree). A higher score corresponded to a high life satisfaction (52). Reliability of internal consistency in this study at baseline and after 16 weeks was α = 0.84 and α = 0.81, respectively, showing a good internal consistency for the SWLS.

Lastly, the Perceived Stress Scale [PSS; (54, 55)] was also used to measure the participant's life situations, assessed as stressful, during the previous month. Seven out of the 13-items was considered negative and 6 as positive, rated on a 5-point Likert scale (0 = never and 4 = very frequently). Items 4–7, 9, 10 and 13 were inverted to calculate the final score. Final scores range between 0 and 52 points, with a higher score representing higher stress (54, 55). Reliability of internal consistency in this study at baseline and 16 week later was α = 0.72 and α = 0.83, respectively, showing a good internal consistency for the PSS.

#### PA and sedentary time assessment

The PA levels (light-, moderate-to-vigorous intensity) and sedentary behavior were measured before the study started and 16 weeks later using a triaxial accelerometer (Actigraph wGT3X+, Actigraph Corporation, Florida, USA). Each study participant received the wGT3X+ accelerometer and a detailed explanation regarding its use. Study participants wore accelerometers on their waist during all waking hours, for seven consecutive days. Participants were instructed to only remove the accelerometer for sleeping and water activities. Additionally, each participant received an activity diary to report their daily bed and waking times moreover to record when and why the accelerometer was removed. These records allowed for a better interpretation and analysis of the accelerometer data. Data were processed using the ActiLife software V6 13.3 (ActiGraph, Florida, US), and raw data were reintegrated into 60-second epochs. The Troiano et al. (56) cut points and wear time validation criteria were used. The accelerometer data were considered valid for a minimum of 4 days (i.e., 3 weekdays and 1 weekend) with 600 min of wear time per day.

#### Assessment of dietary intake

The dietary intake of the participants was assessed using the semi-quantitative Food Frequency Questionnaire (FFQ) (57, 58). The FFQ comprises 8 food groups and frequency consumption with 9 qualitative options (varying from “never or less than once a month” to “6 or more times per day”). The conversion of food into nutrients was performed by specialized nutritionists, using the Food Processor Plus program (ESHA Research, Salem, Oregon, version 11.1) software as a basis, and with nutritional information from US Department of Agriculture food composition tables, adapted to typical Portuguese food (such as, olive, codfish, “feijoada”) (https://portfir-insa.min-saude.pt/).

#### Antibodies against SARS-CoV-2-S1-RBD protein

SARS-CoV-2 infection can manifest itself in different ways, ranging from asymptomatic to mild- or moderate- respiratory and/ or non-respiratory symptoms, as well as severe pneumonia and multiorgan failure (59). SARS-CoV-2 infection has also been linked to a number of long-term problems (called “the post-COVID syndrome”) (59). During this study intervention, none of our study participants reported symptoms of SARS-CoV-2. However, on the premise that not all individuals have symptoms of SARS-CoV-2 infection, laboratory tests were performed to detect the presence or absence of IgG antibodies against SARS-CoV-2. The purpose was to confirm if any of the participants had been infected with the virus during the study, and if so, how this might affect the study outcomes. Serum samples (16-week follow-up samples) were used to qualitative detection of IgG antibodies against the SARS-CoV-2 receptor binding domain (RBD). The ELISA test system E 111-IVD developed by Mediagnost (Reutlingen, Germany) was applied according to the manufacturer's protocol (https://mediagnost.de/en/anti-SARS-CoV-2-elisa/). The test is considered valid if a P/N ratio was > 5. The cut-off is calculated 3x and 5x mean values of negative controls. Thus, values under 3x cut-off are considered negative and values above 5x are considered positive, (i.e., contain anti-SARS-CoV-2-S1 RBD antibodies). Assays were performed and results calculated according to the manufacturer's protocol.

### Statistical analysis

Data are expressed as mean followed by standard deviation (SD) for continuous variables, and as frequency and percentage for categorical variables. The assumption of normality was checked through the z-values from the skewness and kurtosis tests and using visual inspection of the histograms and normal probability plots (P-P plot). The Shapiro-Wilk test and Levene's test were also used to confirm normal distribution and homogeneity of variances. Assuming data normality, the student's independent *T*-test and chi-square test were used to identify differences between the control and CT group at baseline. In case of non-normality of the data, an equivalent non-parametric test was used. Based on our aim, a per-protocol analysis was performed considering only those participants who completed the exercise program. A two-way analysis of variance (ANOVA) for repeated measures was used for intra- and inter-group comparisons. When a *F*-ratio was significant (i.e., *p* ≤ 0.05), the Bonferroni's *post-hoc* test was used to identify mean differences. Log transformation was applied to the outcomes whenever necessary to achieve a normal distribution of the data. For a better interpretation of the data, the values were back transformed from the log scale for presentation in the results section. To compare within groups changes (baseline and 16 weeks later) the magnitude of the effect was calculated using Cohen's *d* effect size and was interpreted as follows: < 0.20 (small), 0.20–0.79 (moderate) and > 0.80 (large) (60). Pearson and Spearman correlation coefficients were also calculated to study the associations between changes (Δ) in the metabolic profile (glycemic and lipid profile), and those in body composition, CRF, and dietary intake variables. The strength of the correlation was classified as follows (61): 0.10–0.30 (little), 0.30–0.50 (low), 0.50–0.70 (moderate), 0.70–0.90 (high) and 0.90–1.00 (very high). The coefficient of determination (r^2^) was also calculated. Data analyses were performed using the SPSS Statistics version 27.0 (SPSS Inc., IBM Company, Chicago, Illinois, USA). GraphPad Prism 9.0 software (GraphPad Software, San Diego, CA, USA) was used for plotting graphs. Significance level was set at *p* ≤ 0.05.

## Results

Thirty-one sedentary middle aged study participants (48.26 ± 7.89 years old) successfully completed the study with 12 in the CT group and 19 in the control group. No adverse events were identified during the intervention. Table 1 presents the baseline characteristics of all study participants and then by group. There were no statistical differences in anthropometric, demographic, dietary intake, CRF, and PA characteristics between groups at baseline. The serum results of the IgG antibodies against the SARS-CoV-2 showed that all participants presented a value lower than OD 0.484, [i.e., anti-SARS-CoV-2 S1 (RBD)] indicating antibodies were not detectable (mean ± SD: 0.266 ± 0.144 units).

**Table 1:** Baseline characteristics of all the study participants and after their randomization into control or the combined training protocol.

**Variables**	**All**	**Control group**	**Combined training group**	***p-*value**
	**(*n* = 31)**	**(*n* = 19)**	**(*n* = 12)**	**between group**
Age, years	48.26 ± 7.89	49.32 ± 7.13	46.58 ± 9.02	0.356
**Women**, ***n*** **(%)**	24 (77.4)	15 (78.9)	9 (75.0)	0.798
Menopausal, *n* (%)	10 (32.3)	6 (31.6)	4 (33.3)	0.831
Married, n (%)	15 (48.4)	10 (52.6)	5 (41.7)	0.222
**Medical history**				
Hypertension, *n* (%)	8 (25.8)	6 (31.6)	2 (16.7)	0.273
Dyslipidaemia, *n* (%)	2 (6.5)	1 (5.3)	1 (8.3)	0.796
Asthma, *n* (%)	2 (6.5)	2 (10.5)	0 (0)	0.284
Regular medication, *n* (%)	12 (38.7)	8 (42.1)	4 (33.3)	0.625
Current-smoking, *n* (%)	2 (6.5)	0 (0)	2 (16.7)	0.066
**Morphological parameters, mean** **±SD**
Body mass (kg)	72.27 ± 15.36	74.12 ± 16.21	69.33 ± 14.07	0.407
Height (cm)	160.49 ± 8.46	159.81 ± 8.96	161.57 ± 7.86	0.581
BMI (kg/m^2^)	27.83 ± 4.26	28.75 ± 4.29	26.37 ± 3.93	0.131
Waist circumference (cm)	93.94 ± 11.99	95.59 ± 11.30	91.32 ± 13.06	0.343
Hip circumference (cm)	105.89 ± 10.91	106.68 ± 11.27	104.63 ± 10.68	0.619
**Sedentary behavior and PA levels**
Valid days (days) #	6.39 ± 0.92	6.53 ± 0.77	6.17 ± 1.12	0.535
Wear time (min/day)	808.95 ± 67.35	811.55 ± 74.02	804.85 ± 58.11	0.793
Sedentary time (min/day)	486.01 ± 88.11	469.68 ± 101.25	511.86 ± 56.64	0.148
LPA (min/day)	305.34 ± 93.97	324.54 ± 105.35	274.94 ± 65.39	0.156
MVPA (min/day) ^a^	16.54 ± 10.69	15.59 ± 11.31	18.05 ± 9.88	0.418
**Dietary intake**				
Energy intake (kcal/day)	2,098.68 ± 773.79	2,092.7 ± 723.0	2,108.2 ± 881.8	0.958
Fat intake (g/day)	84.17 ± 36.46	83.1 ± 32.1	85.8 ± 43.9	0.847
Carbohydrate intake (g/day)	239.48 ± 95.05	236.7 ± 87.3	244.0 ± 110.2	0.839
Protein intake (g/day)	103.52 ± 43.77	106.1 ± 47.7	99.4 ± 38.3	0.683
**Cardiorespiratory fitness**
VO_2max_ (mlo_2_/kg/min) #	31.72 ± 6.46	29.87 ± 5.10	34.48 ± 7.49	0.108

Data are expressed as mean ± standard deviation, count, or percentage as appropriate. Abbreviations: n, number; %, percentage; kg, kilograms; cm, centimeters; m, meters; BMI, body mass index; LPA, light physical activity; MVPA, moderate-to-vigorous physical activity; VO_2max_, maximal oxygen uptake. ^a^Logarithmic transformation was used for the analysis. ^#^Mann-Whitney test.

The total daily energy and macronutrient intake both at baseline and at the 16 weeks of follow-up are presented in Supplementary Table S1. There were no significant effects for group comparisons, time, or their interaction (*p* > 0.05). This indicates that the daily energy or macronutrient intake were not different between the groups and did not change over the course of the 16-week follow-up period. In relation to sedentary time and PA levels, there was a significant time by group interaction for MVPA (min/day) (Supplementary Table S2). *Post-hoc* analysis showed that MVPA increased significantly by 9.67 min/day (*p* = 0.019, *d* = 0.788 [moderate]) in the CT group, and there was a significant difference between the groups at the 16-week follow-up (*p* = 0.018). No significant differences were found for sedentary time or LPA.

Table 2 presents body composition, lipidic and glycaemic profile outcomes assessed at baseline and at the 16 weeks follow-up by group. In relation to body composition outcomes, there was a significant time by group interaction for WC (*F* = 17.813, *p* < 0.001), HC (*F* = 14.205, *p* = 0.001), WHtR (*F* = 18.521, *p* < 0.001) and WHR (*F* = 7.404, *p* = 0.011), in which improvements were observed for the CT group at post-training. *Post-hoc* analysis showed that WC decreased significantly by −2.43 cm (*p* = 0.010, *d* = −1.32 [large]) in the CT group and increased significantly by 2.29 cm (*p* = 0.030, *d* = 0.642 [moderate]) in the control group. The HC decreased −1.60 cm (*p* = 0.009, *d* = −0.73 [moderate]) in the CT group, while the control group increased 1.17 cm (*p* = 0.016, *d* = 0.628 [moderate]). The WHtR decreased −0.015 cm (*p* = 0.009, *d* = −1.33 [large]) in the CT group, while the control group increased 0.01 cm (*p* = 0.002, *d* = 0.662 [moderate]), with a significant difference between the groups at the 16-week follow-up (*p* = 0.013). Finally, results showed that the WHR increased 0.01 cm in the control group (*p* = 0.029, *d* = 0.484 [moderate]).

**Table 2:** Differences between baseline and after a 16-week follow-up and between groups on body composition, glucose, and lipid profile outcomes calculated with two-way ANOVA for repeated measures.

**Outcome**	**Control group**			**Combined training group**			**Time**		**Group**		**Time x**	
	**(*****n*** = **19)**			**(*****n*** = **12)**			**factor**		**factor**		**Group**	
	**Pre**	**Post**	**Δ_mean_ ±SD**	**Pre**	**Post**	**Δ_mean_ ±SD**	***F***	***p* value**	***F***	***p* value**	***F***	***p* value**
**Body composition**	
Body mass (kg)	74.12 ±16.21	74.68 ± 16.85	0.56 ± 1.85	69.33 ± 14.07	69.16 ± 14.08	−0.18 ± 1.79	0.323	0.574	0.801	0.378	1.182	0.286
BMI (kg/m^2)^	28.75 ± 4.29	28.97 ± 4.69	0.22 ± 0.74	26.37 ± 3.93	26.31 ± 3.87	−0.07 ± 0.71	0.299	0.588	2.59	0.119	1.152	0.292
WC (cm)	95.59 ±11.30	97.88 ± 11.66*	2.29 ± 3.57	91.32 ±13.06	88.90 ± 12.74‡	−2.43 ± 1.83	0.015	0.904	2.261	0.144	17.813	**< 0.001**
HC (cm)	106.68 ± 11.27	107.84 ± 11.44*	1.17 ± 1.86	104.63 ± 10.68	103.03 ± 9.37‡	−1.60 ± 2.19	0.350	0.559	0.739	0.397	14.205	**0.001**
WHtR	0.60 ± 0.06	0.61 ± 0.06*†	0.01 ± 0.02	0.56 ± 0.07	0.55 ± 0.07‡	−0.015 ± 0.11	0.006	0.941	4.281	**0.048**	18.521	**< 0.001**
WHR	0.90 ± 0.07	0.91 ± 0.07*	0.01 ± 0.02	0.87 ± 0.07	0.86 ± 0.08	−0.01 ± 0.02	0.020	0.888	1.981	0.170	7.404	**0.011**
BF (%)	37.71 ± 7.40	37.05 ± 7.90	−0.65 ± 1.48	34.42 ± 7.85	34.13 ± 7.88	−0.29 ± 1.37	3.171	0.085	1.198	0.283	0.463	0.501
FM (kg)	28.13 ± 8.82	27.95 ± 9.50	−0.18 ± 1.62	24.24 ± 9.01	23.98 ± 8.88	−0.27 ± 0.10	0.726	0.401	1.384	0.249	0.028	0.868
SMM (kg)	25.30 ± 6.33	25.71 ± 6.53	0.41 ± 0.62	24.95 ± 5.40	24.97 ± 5.46	0.02 ± 1.00	2.215	0.147	0.060	0.808	1.883	0.181
FFM^a^ (kg)	45.89 ±10.99	46.57 ± 11.17	0.68 ± 1.07	45.09 ± 8.91	45.18 ± 8.10	0.09 ± 1.56	2.190	0.150	0.041	0.841	1.300	0.264
**Glucose metabolism**	
Fasting glucose^a^ (mg/dL)	89.89 ±12.40	94.63 ± 7.88*	4.74 ± 10.16	89.00 ± 11.85	92.33 ± 4.91	3.33 ± 9.61	6.341	**0.018**	0.223	0.641	0.119	0.732
Fasting insulin^a^ (mU/L)	8.02 ± 1.58	9.28 ± 3.61	1.26 ± 2.77	7.55 ± 1.15	7.47 ± 0.94	−0.08 ± 0.91	1.770	0.194	2.139	0.154	2.310	0.139
HOMA-IR index^a^	1.79 ± 0.46	2.18 ± 0.88*	0.41 ± 0.76	1.66 ± 0.38	1.70 ± 0.23	0.04 ± 0.31	4.389	**0.045**	2.137	0.155	1.765	0.194
**Lipid Profile**	
TC (mg/dL)	191.21 ± 33.92	192.42 ± 24.47	1.21 ± 23.28	198.50 ± 33.29	192.25 ± 35.92	−6.25 ± 24.81	0.328	0.571	0.109	0.744	0.718	0.404
HDL-C^a^ (mg/dL)	49.89 ± 9.81	50.47 ± 9.42	0.58 ± 6.15	57.08 ± 14.72	55.83 ± 13.86	−1.25 ± 5.72	0.092	0.763	2.299	0.140	0.685	0.414
LDL-C (mg/dL)	122.58 ± 26.86	122.07 ± 19.46	−0.51 ± 19.84	122.10 ± 27.16	116.65 ± 33.94	−5.45 ± 22.17	0.606	0.443	0.108	0.745	0.417	0.523
LDL/HDL ratio	2.51 ± 0.58	2.48 ± 0.49	−0.03 ±0.38	2.25 ± 0.69	2.25 ± 0.94	−0.004 ± 0.41	0.062	0.805	1.085	0.306	0.033	0.858
Triglycerides (mg/dL)	93.68 ±30.44	99.37 ±31.67	5.68 ±32.51	96.58 ± 36.72	99.25 ± 38.01	2.67 ± 17.76	0.661	0.423	0.015	0.903	0.086	0.771

Data are expressed as mean ± standard deviation. ^a^Logarithmic transformation was used for the analysis. BMI, body mass index, WC, waist circumference, HC, hip circumference, WHtR, waist-to-heigh ratio, WHR, waist-to-hip ratio, BF, total body fat, FM, fat mass, SMM, skeletal muscle mass, FFM, fat free mass, HOMA-IR, homeostatic model assessment insulin resistance, TC, total cholesterol, HDL-C, High-density lipoprotein cholesterol, LDL-C, low-density lipoprotein cholesterol, LDL/HDL, low-density lipoproteins/high-density lipoproteins ratio. p ≤ 0.05, ^*^control group pre × control group post; ‡exercise group pre × exercise group post, †control group post × exercise group post. Bold p values mean significant differences.

Furthermore, a significant effect of time was observed in the fasting glucose levels (*F* = 6.341, *p* = 0.018) and the HOMA-IR index (*F* = 4.389, *p* = 0.045) (Table 2). *Post-hoc* analysis showed that fasting glucose and HOMA-IR index increased significantly by 4.74 mg/dL (*p* = 0.029; *d* = 0.466 [moderate]) and 0.41 (*p* = 0.010; *d* = 0.522 [moderate]), respectively, after a 16-week follow up in the control group, compared to baseline. Fasting insulin levels show no effect of time (*F* = 1.770, *p* = 0.194) or interaction (*F* = 2.310, *p* = 0.139). No significant differences were found for the CT group, regarding the glycaemic profile (Table 2). The significant increase in glucose and HOMA-IR index observed in the control group did not differ depending on the BMI status of the participants (*p* > 0.05) (Supplementary Table S3, Supplementary Figure S1). Moreover, there was no effect of either time (for both groups) or group interaction (*p* > 0.05) for total cholesterol, LDL-C, HDL-C, LDL/HDL ratio, and triglycerides (Table 2). In addition, most of our findings (i.e., body composition, HOMA-IR, and lipid profile) did not change after performing an analysis of variance (ANCOVA) including the menopausal status of women as a possible confounding factor (Supplementary Table S4).

**Figure 3:**
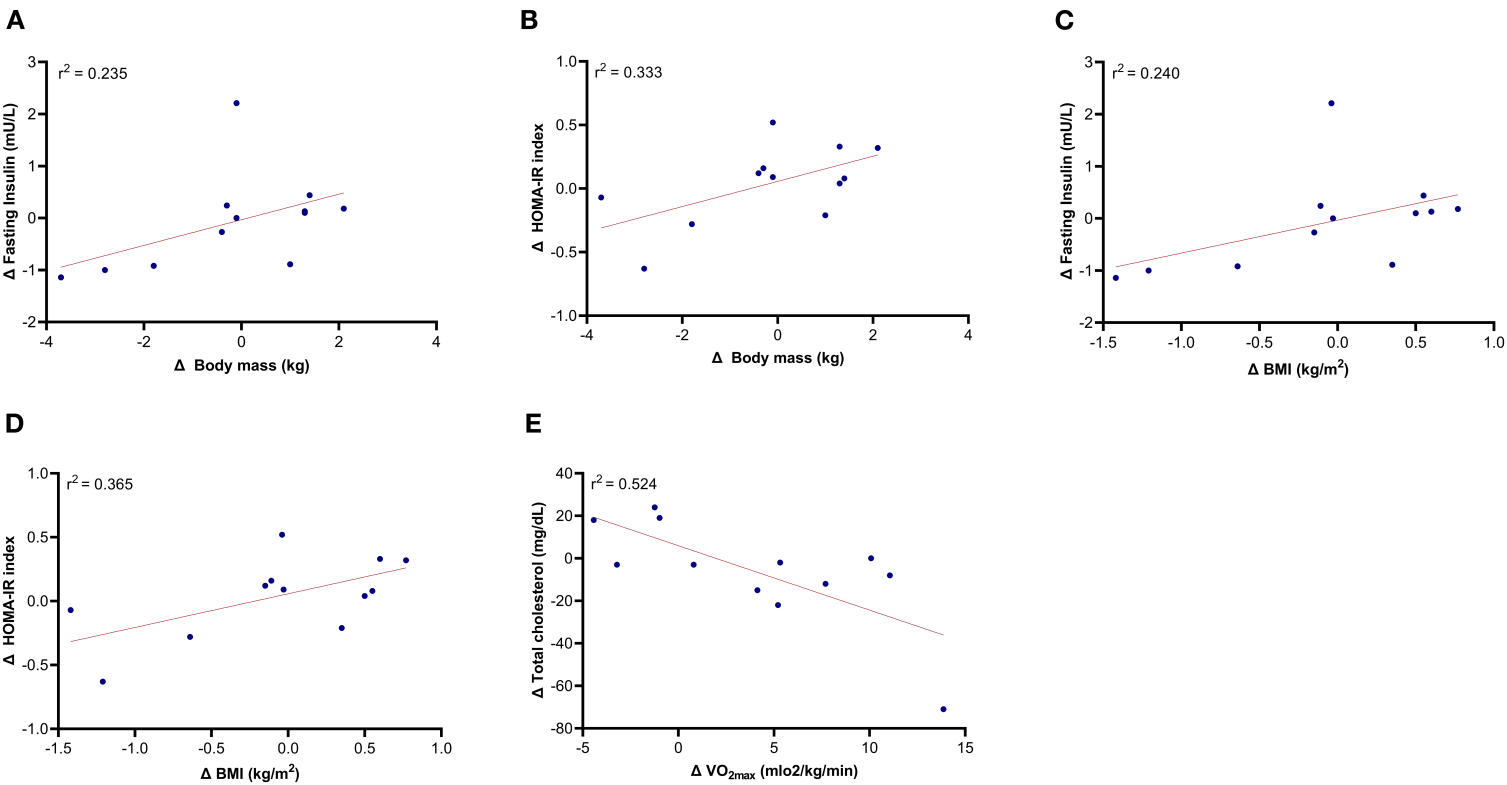
Relationship of the changes in fasting insulin, HOMA-IR, total cholesterol with the changes in body composition and cardiorespiratory fitness (excluding control group). **(A)** Δ Body mass and Δ fasting insulin; **(B)** Δ Body mass and Δ HOMA-IR index; **(C)** Δ BMI and Δ fasting insulin; **(D)** Δ BMI and Δ HOMA-IR index; **(E)** Δ Vo2max and Δ Total cholesterol.

A significant positive relationship was detected between the change in body mass and the change in fasting insulin (*r* = 0.704, *p* = 0.011 [high]; Figure 3A) and HOMA-IR index (*r* = 0.577, *p* = 0.050 [moderate]; Figure 3B). Moreover, changes in BMI were positively associated with changes in insulin (*r* = 0.643, *p* = 0.024 [moderate]; Figure 3C) and HOMA-IR (*r* = 0.605, *p* = 0.037 [moderate]; Figure 3D). CRF was negatively correlated with total cholesterol (*r* = −0.578, *p* = 0.049 [moderate]; Figure 3E). No significant correlations were found between changes in metabolic profile outcomes and other parameters of body composition and dietary pattern variables.

Table 3 shows the results obtained for stress and HRQoL. Regarding the salivary stress hormones (cortisol and α-amylase hormones), there were no significant effects of time, group, or interaction (time x group) (*p* > 0.05). However, there was a main effect of group for the PSS score (*F* = 5.399, *p* = 0.027), which is represented by a significant difference of 5.5 points (*p* = 0.025) between the groups at the 16-week follow-up. Regarding to HRQoL, a significant effect of time was found in satisfaction with life (*F* = 5.561, *p* = 0.025). Life satisfaction increased 3.17 points in the CT group (*p* = 0.038, *d* = 0.690 [moderate]) after the exercise intervention. No significant effect of time was verified in the control group. In relation to WHOQOL-Bref questionnaire, we observed a main effect of group to the physical health domain (*F* = 7.027, *p* = 0.013), psychological health domain (*F* = 4.552, *p* = 0.041) and environmental health domain (*F* = 10.59, *p* = 0.003). These results represent differences between groups at the 16-weeks follow-up, with better values observed in the CT group.

**Table 3:** Differences between baseline and 16-week follow-up and between groups on the salivary stress hormones and HRQoL, calculated with two-way ANOVA for repeated measures.

**Outcome**	**Control group**			**Combined training group**			**Time**		**Group**		**Time x**	
	**(*****n*** = **19)**			**(*****n*** = **12)**			**factor**		**factor**		**Group**	
	**Pre**	**Post**	**Δ_mean_ ±SD**	**Pre**	**Post**	**Δ_mean_ ±SD**	***F***	***p* value**	***F***	***p* value**	***F***	***p* value**
**Stress levels**
Cortisol (μg/mL)	0.33 ± 0.18	0.28 ± 0.12	−0.05 ± 0.21	0.34 ± 0.15	0.28 ± 0.16	−0.06 ± 0.21	2.071	0.161	0.072	0.790	0.015	0.903
Alpha-amylase^a^ (U/mL)	46.04 ± 29.38	34.97 ± 17.86	−11.07 ± 5.68	58.57 ± 35.78	53.19 ± 25.28	−5.38 ± 18.35	2.011	0.167	3.447	0.074	0.847	0.365
PSS (score)	23.95 ± 3.73	24.58 ± 5.39†	0.63 ± 6.21	21.67 ± 6.30	19.08 ± 7.61	−2.58 ± 7.45	0.622	0.437	5.399	**0.027**	1.689	0.204
**HRQoL**
SWLS (score)	15.89 ± 4.61	17.11 ± 4.15†	1.11 ± 5.29	17.0 ± 3.74	20.17 ± 2.48‡	3.17 ± 4.59	5.561	**0.025**	3.392	0.076	1.111	0.301
**WHOQOL-BREF (Score)**
Physical health (0–100)	64.66 ± 17.04	63.91 ± 12.42†	−0.75 ± 11.58	73.80 ± 11.83	79.94 ± 12.66	6.13 ± 9.34	1.830	0.187	7.027	**0.013**	2.998	0.094
Psychological health (0–100)	63.16 ± 17.64	61.62 ± 11.9†	−1.53 ± 14.38	70.14 ± 11.49	74.31 ± 14.52	4.17 ± 13.53	0.258	0.615	4.552	**0.041**	1.209	0.281
Social relationship (0–100)	72.72 ± 12.54	70.18 ± 9.28	−2.54 ± 13.98	71.53 ± 18.28	77.08 ± 13.82	5.56 ± 15.21	0.319	0.577	0.489	0.490	2.307	0.140
Environmental health (0–100)	60.36 ± 9.99	61.02 ± 10.89†	0.66 ± 10.45	68.75 ± 10.23	73.70 ± 9.28	4.95 ± 10.27	2.145	0.154	10.59	**0.003**	1.256	0.272

Data are reported as mean ± SD. ^a^Logarithmic transformation was used for the analysis. PSS, perceived stress scale; SWLS, satisfaction with life scale; HRQoL, health-related quality of life. p ≤ 0.05, ^*^control group pre × control group post; ‡exercise group pre × exercise group post, †control group post × exercise group post. Bold p values mean significant differences.

## Discussion

This study aimed to analyse the effects of a CT program performed during the first national lockdown due to the COVID-19 pandemic on body composition, metabolic profile, quality of life, and stress in sedentary middle-aged workers. Additionally, we examined whether changes in the metabolic profile were associated with changes in health-related outcomes which are modifiable by exercise. Our results showed that a 16-week CT program reduced abdominal adiposity and improved HRQoL, while no significant changes were noted in either glycaemic or lipid profile parameters (i.e., CT participants were able to maintain their metabolic parameters). In addition, changes in body mass and BMI were positively correlated with changes in fasting insulin and HOMA-IR. Also, changes in CRF were negatively associated with changes in total cholesterol. Instead, after a period of 16 weeks, the non-exercise control group increased waist and hip circumferences, progressed into insulin resistance, as shown by the increase in glucose and HOMA-IR and had higher perceived stress levels and lower HRQoL, when compared to the CT group.

It has been previously reported that CT is an effective tool to reduce body mass and FM while increasing SMM in sedentary adults (23, 24). The study of Sillanpää et al. (24) observed that 21-weeks of CT markedly enhanced body composition, with a significant decrease of −4.8% in total FM and an increase of 2.2% and ~3.0% in SMM of the arms and legs, respectively, in middle-aged and older women. These results agree with those obtained by Amaro-Gahete et al. (23), that found that 12-weeks of CT result in a significant decrease of −4% in FM and an increase of 4% in SMM, in middle-aged sedentary adults. However, in the current study, the CT program significantly decreased WC, HC and WHtR, without any significant change in other morphologic outcomes, such as SMM and FFM. It is possible that the low resistance training loads used in the last weeks of the program were not enough to promote SMM improvement. Nevertheless, other studies with low- to medium- training intensities also did not find significant changes in SMM after 12-weeks of CT in adults with the metabolic syndrome (aged 48–77 years old) (22) and obesity (aged 35–55 years old) (62).

The CT has also been positioned as a promising tool to ameliorate metabolic health, through the management of glycaemic and lipid profiles. In a study, Amanat et al. (27) observed that 12-weeks of CT exercise reduced insulin resistance, fasting insulin, glucose, triglycerides, LDL-C, and total cholesterol in overweight women (aged 46–60 years old) with the metabolic syndrome. Another study (28) also found significant improvements in fasting glucose, HbA1c, and HOMA-IR after 12-weeks of CT program in women aged over 45 years at high risk of T2DM. Similarly, Amaro-Gahete et al. (26) found that 12-weeks of CT significantly improved HDL-C, total cholesterol, and insulin sensitivity in sedentary middle-aged adults (aged 40 to 65 years old). Also, Sillanpää et al. (24) observed that 21-weeks of CT decreases serum fasting insulin in women with 39 to 64 years old, whereas no significance was observed in other metabolic outcomes. Our study findings partially disagree with those previously reported, since the present CT program did not promote any significant change in glycaemic (i.e., fasting insulin, glucose, or HOMA-IR) or lipid (total cholesterol, HDL-C, LDL-C, LDL/HDL, and triglycerides) profiles. Furthermore, there were also no significant differences between the CT group and the non-exercise control group in these outcomes. However, it is important to note that while the control group progressed into insulin resistance [HOMA-IR above 2.0 (42, 43)] during the 16-week period, the CT program was important to prevent this group from progressing to an insulin resistant phenotype.

The different intervention contexts could be a potential explanation for the discrepancies between our results and the results of the mentioned studies (i.e., our exercise program was conducted at home in the context of movement restrictions, and there was a drastic alteration in the CT program within the middle of the intervention due to lockdowns). Moreover, it is important to note that some of the studies mentioned above included participants with associated comorbidities (27, 28). Our findings could also be explained by the lack of changes in SMM since this outcome may also play a beneficial role in whole-body glucose homeostasis and metabolic health (11, 63).

But certainly, the most plausible reason can be associated with the changes in daily people's lives during the COVID-19 pandemic-related lockdown. Unfortunately, we only assessed PA levels at the end of the 16-week follow-up, which corresponded to the end of the first lockdown in Portugal. An intermediate assessment would have provided relevant data, as a growing body of evidence showed that the sudden state of lockdown due to the COVID-19 pandemic had a tremendous impact on many aspects of daily life, including changes in behavioral patterns (5–7), modified dietary habits (64), as well increased feelings of distress and anxiety (18). Thus, it is expected that these and other confounding factors may have interfered with the results of this study. Furthermore, despite instructions to maintain the same nutritional pattern over the 16-week intervention, and the auto-reported unchanged (i.e., according to FFQ), people could be more careless with their calorie intake and nutritional quality, believing that exercise will compensate for these differences (22). However, our results agree with those obtained by previous studies that also found no significant changes in the lipid (22, 24, 62) and the glycaemic profile (62, 65) after a CT program in sedentary adults. Further studies are needed to confirm the current results.

Furthermore, we observed that changes in BMI positively predict 24% and 37% of the changes expected for fasting insulin and HOMA-IR, respectively. These findings have important clinical relevance and in part confirm the evidence that FM accumulation is closely associated with the increase of insulin resistance – a major risk marker of impaired glucose metabolism, T2DM, and CVD (8, 11, 66). Evidence suggests that the chronic low-grade inflammation present in adipose tissue is involved in the pathogenesis of insulin resistance (9, 13). Even in young children, a recent study observed lower adiponectin levels, a marker of adiposity secretory dysfunction, and elevated leptin secretion in insulin-resistant children in comparison to lean or obese insulin-sensitive children (67). Other studies in middle-aged adults (first-degree relatives with T2DM and massive obesity) also observed that 30% of them that have increased fat cell size and increased WHR were those that were characterized by insulin resistance (68, 69).

Consequently, it is biologically acceptable that exercise could mitigate the chronic inflammation in adipose tissues by reducing adipose tissue mass and regulating adipokine expression, resulting in enhanced insulin sensitivity (70). According to Eaton and Eaton (11) the insulin sensitivity is directly associated to %SMM but is inversely related to %BF. The mechanisms whereby SMM induces improvements in whole-body glucose homeostasis are not fully understood, however, a recent review suggests that a biological mechanism can be the greater SMM capillarity and its vasodilator response (63).

Moreover, our results suggests that the changes in CRF were associated with a decrease in total cholesterol in the exercise group. Prior research has also shown that CRF improves lipid and lipoprotein profiles (71) through mechanisms that may include increased activity of lipoprotein lipase in active SMM (71–73). This increase leads to a higher triglyceride clearance rate; enhanced HDL-C; and improved lipid and lipoprotein transport from the tissues to the liver (71–73). Moreover, a recent study also shows that higher CRF is associated with decreased probability of clinical high blood pressure and lower insulin resistance in overweight children (74). Total cholesterol, LDL-C, and triglycerides gradually increase until the mid-40's to early 50's, so it seems important to maintain a good CRF level in these ages to prevent and/or delay the manifestation of dyslipidaemia and its related non-communicable diseases (71). Notably, our results are important and show the clinical importance of regular daily exercise, even if performed at home in contexts of movement restrictions, to maintain the glucose and lipid levels and thus delay/prevent the manifestation of metabolic disorders. In contrast, the non-exercise control group that maintained their sedentary lifestyle increased abdominal obesity, fasting glucose, and HOMA-IR. These results agree with several studies showing that a sedentary lifestyle is associated with metabolic derangements such as obesity, insulin resistance, and T2DM (8–12).

In relation to HRQoL, our results showed that the CT group increased their life satisfaction after the exercise program. Furthermore, when compared to the control group, the CT group presented lower perceived stress levels and higher HRQoL in the physical, psychological, and environmental domains at the follow-up. Our findings are consistent with other studies that confirm that the participants who were more physically active were generally more satisfied with their lives (75). Similarly, a review study (76) found that exercise (independently of the type) has a positive effect on the HRQoL of healthy older adults. On the other hand, a sedentary lifestyle is unfavorably associated with perceived stress and HRQoL (15–17, 77). Some studies showed that the imposed stay-at-home orders and other lockdown measures due to the COVID-19 pandemic affected negatively the HRQoL and mental health of the populations (78, 79). This data is alarming since that lower HRQoL has been associated with the development of non-communicable diseases and mental health issues (79, 80). Based on this evidence, it seems that our exercise program was also an important strategy to prevent a decrease in HRQoL and life satisfaction.

Taken together, our findings suggest that the practice of combined exercise for the prevention of metabolic disorders and psychologic conditions are essential in all aspects of management, particularly during the COVID-19 pandemic-related lockdowns. In addition, exercise has also been identified as an effective strategy against the increased hospitalization rates due to respiratory diseases, such as the related COVID-19 comorbidities (81). Conversely, chronic physical inactivity and sedentary behavior is associated with a higher risk of COVID-19 hospitalization, independently of age, sex, smoking, alcohol consumption, and obesity (82). These data are clinically relevant, especially nowadays, where thousands of people continue to be affected daily by the SARS-CoV-2 virus infection.

There are three important limitations to this study that should be considered when interpreting the findings. First, the insulin resistance was not determined using the golden standard, the hyperinsulinemia euglycemic glucose (HIEG) clamp technique. However, previous studies have shown that the HOMA-IR method is recognized and validated method to determine insulin resistance (83). Second, with the beginning of the pandemic-related lockdown, the exercise program underwent some changes, i.e., it was impossible to continue with the progression of loads in the resistance training as initially planned. Third, other residual confounding factors such as teleworking period, dietary pattern, sedentary and physical activity levels during the home-confinement period and other unknown factors, may have cofounded some of the results. Additionally, potential covariates such as the menopausal status of the women may also have confounded some of the results. However, we tried to mitigate this limitation by adopting specific statistical procedures, considering the menopausal status as a confounding variable.

The findings of the present study should be analyzed in the context of home-confinement due to the COVID-19 lockdown. We suggest that future experimental and longitudinal studies could be carried to confirm these results. Moreover, given the current COVID-19 pandemic, it is essential that future PA guidelines encourage the practice of PA/physical exercise, and integrate specific guidelines for home-based exercise.

## Conclusion

The findings of the present study suggest that the participants who remained physically active through a supervised exercise program, during the first pandemic-related lockdown, were able to mitigate the deleterious effects associated with a sedentary lifestyle. Specifically, our results showed that a 16-week CT program helped maintain glucose and lipid levels, reduced abdominal adiposity, and improved HRQoL. In contrast, the non-exercise control group participants who remained physically inactive increased abdominal obesity, progressed into insulin resistance (as shown by the increase in fasting glucose and HOMA-IR), and had higher perceived stress levels and lower HRQoL when compared to the exercise group. Despite the inherent limitations, our findings have important clinical implications. They suggest that a CT program, even if performed at home in the context of movement restrictions, could be an effective and cost-efficient strategy to prevent metabolic disorders and mental health problems among sedentary workers.

## Data availability statement

The original contributions presented in the study are included in the article/Supplementary material, further inquiries can be directed to the corresponding authors.

## Ethics statement

The studies involving human participants were reviewed and approved by Ethical Committee for Health of the Faculty of Sport Sciences and Physical Education, University of Coimbra (reference: CE/FCDEF-UC/00512019). The patients/participants provided their written informed consent to participate in this study.

## Author contributions

JF, AT, PD-M, and FS contributed to the conception and design of the study. FS, CF, and CS performed the experiments. FS and RR analyzed and interpreted data. JF, AT, and PD-M supervised the work. FS drafted the manuscript. JF, AT, PD-M, CS, RP, JS, EC, and AM critically reviewed the contents of the manuscript. All authors have read and approved the submitted version of the manuscript.

## Funding

FS was a grant holder from the Portuguese Foundation for Science and Technology (2020.08759.BD). The funder had no role in the development and preparation of the manuscript.

## Conflict of interest

The authors declare that the research was conducted in the absence of any commercial or financial relationships that could be construed as a potential conflict of interest.

## Publisher's note

All claims expressed in this article are solely those of the authors and do not necessarily represent those of their affiliated organizations, or those of the publisher, the editors and the reviewers. Any product that may be evaluated in this article, or claim that may be made by its manufacturer, is not guaranteed or endorsed by the publisher.
