# Supplementary material for: Effects of combined training during the COVID-19 pandemic on metabolic health and quality of life in sedentary workers: A randomized controlled study
Source: Front Public Health. 2022 Nov 10;10:1040714. doi: 10.3389/fpubh.2022.1040714 (PMC9686330; doi:10.3389/fpubh.2022.1040714)
Supplement: Supplementary file 1 [file Data_Sheet_1.pdf]

Supplementary Material

Supplementary Table S1. Dietary intake at baseline and after 16 weeks of intervention.

Outcome	Control group (n=19)			Combined training group (n=12)			Time factor		Group factor		Time x Group	
	Pre	Post	Δ ± SD	Pre	Post	Δ ± SD	*F*	*p*-value	*F*	*p*-value	*F*	*p*-value
Energy intake (kcal/day)	2092.7 ± 723.0	2095.0 ± 861.5	-2.35 ± 777.41	2108.2 ± 881.8	1751.2 ± 680.8	-356.9 ± 684.7	1.672	0.206	0.405	0.530	1.717	0.200
Fat intake (g/day)	83.1± 32.1	89.61 ±40.0	6.5 ± 41.2	85.8 ± 43.9	72.8 ± 28.2	-12.9 ± 34.5	0.205	0.654	0.381	0.542	1.842	0.185
Carbohydrate intake (g/day)	236.7 ± 87.3	233.1 ± 95.6	-3.53 ± 84.9	244.0 ± 110.2	192.0 ± 78.3	-52.0 ± 93.0	2.923	0.098	0.314	0.580	2.226	0.147
Protein intake (g/day)	106.1 ± 47.7	94.1 ± 41.6	-12.0 ± 39.34	99.4 ± 38.3	85.6 ± 33.5	-13.8 ± 45.5	2.812	0.104	0.333	0.569	0.014	0.906

Notes: Data are expressed and mean ± standard deviation.

Supplementary Table S2. Sedentary behavior and PA levels at baseline and after 16 weeks of intervention.

**Outcome**	**Control group (n=19)**			**Combined training group (n=12)**			**Time factor**		**Group factor**		**Time x Group**	
	Pre	Post	Δ ± SD	Pre	Post	Δ ± SD	*F*	*p*-value	*F*	*p*-value	*F*	*p*-value
Sedentary time (min/day)	469.68±101.25	468.37±111.20	-1.31±90.12	511.86±56.64	486.95±48.72	-24.91±66.56	0.752	0.393	1.061	0.312	0.610	0.441
LPA (min/day)	324.54±105.35	325.42±106.88	0.88±72.32	274.94±65.39	276.12±78.93	1.18±74.58	0.006	0.940	2.353	0.136	0.000	0.991
MVPA (min/day) [a]	15.59±11.31	14.65±7.79†	-0.94±8.64	18.05±9.88	27.73±14.96	9.67±12.28	2.97	0.096	3.54	0.070	4.68	**0.039**
Wear time (min/day)	811.55±74.02	808.44±62.56	-3.10±71.11	804.85±58.11	790.80±57.64	-14.05±81.70	0.382	0.541	0.394	0.535	0.156	0.696

Notes: Data are expressed as mean ± standard deviation. Abbreviations: LPA, light physical activity; MVPA, moderate-to-vigorous physical activity. [a] Logarithmic transformation was used. $p \leq 0.05$, †control group post × exercise group post. Bold *p* values mean significant differences for the analysis.

Supplementary Table S3. Changes in glucose and HOMA-IR index in the control group participants, stratified based on their BMI status (normal weight [18.5-24.9 kg/m^2], overweight [25.0-29.9 kg/m^2] and obesity [30.0-35.0 kg/m^2]).

Outcome	Normal weight (n = 4)			Overweight (n = 7)			Obesity (n = 8)			Time factor		Group factor		Time x Group	
	Pre	Post	Δ ± SD	Pre	Post	Δ ± SD	Pre	Post	Δ ± SD	*F*	*p*-value	*F*	*p*-value	*F*	*p*-value
Fasting glucose (mg/dL)	89.50 ± 9.98	87.75 ± 9.07	-1.75 ± 3.78	91.57 ± 18.33	97.29 ± 6.37	5.71 ± 13.14	88.63 ± 7.75	95.75 ± 7.34	7.13 ± 8.94	2.321	0.147	0.493	0.620	1.078	0.364
HOMA-IR index[a]	1.72 ± 0.54	2.03 ± 1.19	0.31 ± 0.69	1.95 ± 0.65	2.25 ± 0.53	0.31 ± 0.38	1.68 ± 0.11	2.20 ± 1.06	0.52 ± 1.07	4.040	0.062	0.396	0.679	0.145	0.866

Notes: Data are expressed as mean ± standard deviation. [a] Logarithmic transformation was used for the analysis. Abbreviations: HOMA-IR, homeostatic model assessment insulin resistance. $p \leq 0.05$ mean significant differences.

Supplementary Figure S1. Changes in glucose and HOMA-IR index in the control group participants, stratified based on their BMI status. **(a)** Fasting glucose (mg/dL), **(b)** HOMA-IR index.

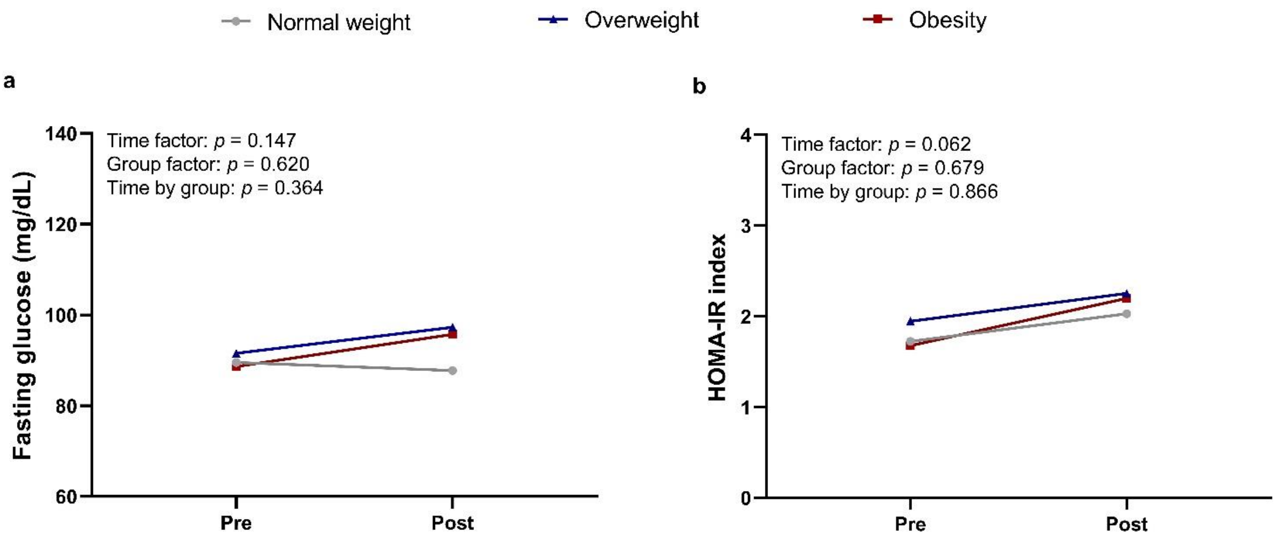

Supplementary Table S4. Changes on the body composition, glucose, and lipid profile outcomes adjusted for menopausal status of women as cofounding factor.

Outcome	Time factor		Group factor		Time x Group	
	F	*p* value	*F*	*p* value	*F*	*p* value
Body Composition						
Body mass (kg)	0.003	0.959	0.046	0.832	0.652	0.429
BMI (kg/m^{2})	0.000	0.994	0.721	0.406	0.653	0.428
WC (cm)	0.089	0.768	0.952	0.340	16.117	**0.001***‡
HC (cm)	0.006	0.937	0.131	0.721	16.727	**0.001***‡
WHtR	0.128	0.724	2.279	0.146	16.645	**0.001***‡
WHR	0.108	0.746	3.119	0.092	5.244	**0.032***
BF (%)	0.089	0.769	0.439	0.515	0.294	0.593
FM (kg)	0.132	0.720	0.207	0.654	0.132	0.720
SMM (kg)	0.179	0.676	0.027	0.871	0.628	0.437
FFM[a] (kg)	0.117	0.735	0.036	0.851	0.601	0.447
Glucose metabolism						
Fasting glucose[a] (mg/dL)	2.460	0.132	0.201	0.658	0.002	0.968
Fasting insulin[a] (mU/L)	6.035	**0.023***	0.251	0.622	1.723	0.203
HOMA-IR index[a]	7.702	**0.011***	0.371	0.549	1.064	0.314
Lipid Profile						
TC (mg/dL)	1.239	0.278	0.384	0.542	0.238	0.631
HDL-C[a] (mg/dL)	0.022	0.885	2.026	0.169	0.003	0.954
LDL-C (mg/dL)	2.430	0.134	2.783	0.110	0.236	0.632
LDL/HDL ratio	2.118	0.160	3.643	0.070	0.230	0.637
Triglycerides (mg/dL)	0.604	0.446	0.060	0.809	0.112	0.741

Notes: Data are expressed as mean ± standard deviation. [a] Logarithmic transformation was used for the analysis. Abbreviations: BMI, body mass index, WC, waist circumference, HC, hip circumference, WHtR, waist-to-heigh ratio, WHR, waist-to-hip ratio, BF, total body fat, FM, fat mass, SMM, skeletal muscle mass, FFM, fat free mass, HOMA-IR, homeostatic model assessment insulin resistance, TC, total cholesterol, HDL-C, High-density lipoprotein cholesterol, LDL-C, low-density lipoprotein cholesterol, LDL/HDL, low-density lipoproteins/high-density lipoproteins. $p \leq 0.05$, *control group pre × control group post; ‡exercise group pre × exercise group post. Bold *p* values mean significant differences.
